# The Road of Solid Tumor Survival: From Drug-Induced Endoplasmic Reticulum Stress to Drug Resistance

**DOI:** 10.3389/fmolb.2021.620514

**Published:** 2021-04-13

**Authors:** Shulong Cao, Jingyi Tang, Yichun Huang, Gaofeng Li, Zhuoya Li, Wenqi Cai, Yuning Yuan, Junlong Liu, Xuqun Huang, Haiyuan Zhang

**Affiliations:** ^1^ School of Basic Medicine, Health Science Center, Yangtze University, Jingzhou, China; ^2^ Clinical Medical College, Hubei University of Science and Technology, Xianning, China; ^3^ Edong Healthcare Group, Department of Medical Oncology, Huangshi Central Hospital, Affiliated Hospital of Hubei Polytechnic University, Huangshi, China

**Keywords:** drug resistance, endoplasmic reticulum stress, solid tumor, unfolded protein response, immunosuppression

## Abstract

Endoplasmic reticulum stress (ERS), which refers to a series of adaptive responses to the disruption of endoplasmic reticulum (ER) homeostasis, occurs when cells are treated by drugs or undergo microenvironmental changes that cause the accumulation of unfolded/misfolded proteins. ERS is one of the key responses during the drug treatment of solid tumors. Drugs induce ERS by reactive oxygen species (ROS) accumulation and Ca^2+^ overload. The unfolded protein response (UPR) is one of ERS. Studies have indicated that the mechanism of ERS-mediated drug resistance is primarily associated with UPR, which has three main sensors (PERK, IRE1α, and ATF6). ERS-mediated drug resistance in solid tumor cells is both intrinsic and extrinsic. Intrinsic ERS in the solid tumor cells, the signal pathway of UPR-mediated drug resistance, includes apoptosis inhibition signal pathway, protective autophagy signal pathway, ABC transporter signal pathway, Wnt/β-Catenin signal pathway, and noncoding RNA. Among them, apoptosis inhibition is one of the major causes of drug resistance. Drugs activate ERS and its downstream antiapoptotic proteins, which leads to drug resistance. Protective autophagy promotes the survival of solid tumor cells by devouring the damaged organelles and other materials and providing new energy for the cells. ERS induces protective autophagy by promoting the expression of autophagy-related genes, such as Beclin-1 and ATG5–ATG12. ABC transporters pump drugs out of the cell, which reduces the drug-induced apoptosis effect and leads to drug resistance. In addition, the Wnt/β-catenin signal pathway is also involved in the drug resistance of solid tumor cells. Furthermore, noncoding RNA regulates the ERS-mediated survival and death of solid tumor cells. Extrinsic ERS in the solid tumor cells, such as ERS in immune cells of the tumor microenvironment (TME), also plays a crucial role in drug resistance by triggering immunosuppression. In immune system cells, ERS in dendritic cells (DCs) and myeloid-derived suppressor cells (MDSCs) influences the antitumor function of normal T cells, which results in immunosuppression. Meanwhile, ERS in T cells can also cause impaired functioning and apoptosis, leading to immunosuppression. In this review, we highlight the core molecular mechanism of drug-induced ERS involved in drug resistance, thereby providing a new strategy for solid tumor treatment.

## Introduction

Tumors are divided into solid and nonsolid tumors. The former can form masses that can be visualized by computed tomography, magnetic resonance imaging, and other examination methods. Solid tumor is one of the common causes of death in human beings (Bray et al., 2018). Owing to the decline of body functioning or the missed operation opportunity of patients with advanced solid tumors, the surgical treatment mode for early-stage solid tumors is not suitable for advanced solid tumors. Therefore, drug-centered systemic therapy (including chemotherapy, targeted therapy, biotherapy, and endocrine therapy) has become the mainstream treatment for advanced solid tumors (Ngambenjawong et al., 2017; Coleman et al., 2019; Geurten et al., 2019; Schirrmacher, 2019). In this context, the main problem encountered in the clinical treatment of solid tumors is drug resistance (Radhi, 2016; Baxter et al., 2018; Boumahdi and de Sauvage, 2020), including intrinsic and acquired resistance. Acquired drug resistance leads to solid tumor recurrence and distant metastasis, and is considered to be the major reason for solid tumor therapy failure (Sun et al., 2017; Wu et al., 2020). There is hence an urgency to establish the mechanism of solid tumor resistance, and many studies have made contributions in this regard, for example, investigations on multidrug resistance of transporters in the cell membrane (Liu et al., 2016; Briz et al., 2019; Buondonno et al., 2019) and drug resistance mechanisms based on tumor stem cells (Zhao, 2016; Prieto-Vila et al., 2017). Endoplasmic reticulum stress (ERS), which is a double-edged sword, plays a significant role in regulating the pro-death or pro-survival signals of solid tumor cells (White-Gilbertson et al., 2013). Basic results obtained so far on this topic have suggested (Avril et al., 2017; Bahar et al., 2019) that ERS-mediated tumor cell survival is gradually becoming the key factor in solid tumor drug resistance. In the past years, with the increasing application of drugs and the deepening of research in this domain, many basic reports have been published (Cubillos-Ruiz et al., 2017; Jin et al., 2017; Li L. et al., 2018) on drug resistance mediated by drug-induced ERS, such as intrinsic apoptosis inhibition, protective autophagy, ABC transporters, and extrinsic immunosuppression. However, a systemic report on drug resistance in solid tumor cells has not yet been published. Herein, we have summarized the approaches for ERS induction and unfolded protein response (UPR) sensor activation, and the modes of drug resistance (Table 1). Moreover, we have introduced the mechanism of drug-induced ERS and ERS-mediated intrinsic and extrinsic drug resistance.

**TABLE 1:** Approaches of intrinsic drug-induced ERS causing drug resistance in solid tumors.

Drugs		Solid tumors	Approaches of drug-induced ERS	Activation of UPR sensors	Modes of drug resistance	References
Classification	Sorts					
Alkylating agent	Cisplatin	Lung cancer	Unknown	PERK, IRE1α	Protective autophagy	Shi et al. (2016)
	Oxaliplatin	Colorectal cancer	Unknown	PERK	Drug efflux	Salaroglio et al. (2017)
Antibiotic agents	Doxorubicin	Hepatocellular carcinoma	ROS accumulation	PERK	Apoptosis inhibition/drug efflux	Li L. et al. (2015)
	Salinomycin	Glioma	ROS accumulation	IRE1α	Protective autophagy	Yu et al. (2017)
		Non-small cell lung cancer	Unknown	PERK	Protective autophagy	(Li et al., 2013)
	Tunicamycin	Melanoma	Unknown	ATF6, IRE1α	Protective autophagy	Luan et al. (2015)
Antimetabolites	Methotrexate	Ovarian choriocarcinoma	ROS accumulation	PERK	Protective autophagy	Shen et al. (2015)
	5-Fluorouracil	Hepatocellular carcinoma	Unknown	PERK	Apoptosis inhibition	Liu et al. (2016)
		Breast cancer	Unknown	PERK, IRE1α, ATF6	Proliferation	Yao et al. (2020)
Hormone agents	Tamoxifen	Breast cancer	Unknown	GRP78	Wnt signaling	Pujari et al. (2016)
Immunosuppressive agent	Imiquimod	Melanoma	Ca^2+^ overload	PERK, IRE1α	Apoptosis inhibition	El-Khattouti et al. (2016)
Proteasome agent	Bortezomib	Prostate tumor	Unknown	IRE1α	Wnt signaling	Rodvold et al. (2017)
Tubulin agents and spindle poisons	Pentoxifylline	Melanoma	Ca^2+^ overload	IRE1α	Protective autophagy	Sharma et al. (2016)
Targeted agents	Apatinib	Colorectal cancer	Ca^2+^ overload	IRE1α	Protective autophagy	Cheng et al. (2018)
	Cetuximab	Head and neck squamous cell carcinoma	ROS accumulation	PERK	Protective autophagy	Lei et al. (2016)
Targeted agents	Sorafenib	Hepatocellular carcinoma	Unknown	PERK	Protective autophagy	Zhou et al. (2019)
	Sunitinib	Renal cell carcinoma	Unknown	PERK, IRE1α	NF-κB pro-survival pathway	Makhov et al. (2018)
	Vemurafenib	Thyroid cancer	Unknown	PERK	Protective autophagy	Wang et al. (2017)
Other	AUY922	Colorectal cancer	Unknown	IRE1α	Apoptosis inhibition	Wang et al. (2018)

ATF6, activating transcription factor 6; IRE1α, inositol-requiring enzyme 1 alpha; PERK, protein kinase RNA-like endoplasmic reticulum kinase; NF-κB, nuclear factor “kappa-light-chain-enhancer” of activated B cells; ROS, reactive oxygen species.

## Intrinsic Drug-Induced ERS and Drug Resistance in Solid Tumors

### Drug-Induced ERS in Solid Tumors

Past research (Okubo et al., 2018) has shown that drugs could induce ERS in solid tumor cells. Various antitumor drugs can induce ERS through ROS accumulation (Shen et al., 2015) or Ca^2+^ overload (Sharma et al., 2016; Cheng et al., 2018). Abnormal ROS accumulation is often found in drug-resistant tumor cells (Yu et al., 2017). Drugs can inhibit the mitochondrial electron transport chain (ETC) and cause single electron transfer reactions, which induce the generation of reactive oxygen species (ROS) (Jiang et al., 2019). High ROS often induces the abnormal accumulation of unfolded/misfolded proteins in the lumen of the endoplasmic reticulum (ER) by attacking the free sulfhydryl groups needed for maintaining protein folding and enzyme activity and initiating ERS. Ca^2+^ overload also plays an important role in the occurrence of drug-induced ERS (Cheng et al., 2018). Drugs initiate the Ca^2+^ channels on the tumor cell membrane after entering the cell through the transporters, which causes the influx of extracellular Ca^2+^ (King and Wilson, 2020). Ca^2+^ is released from the ER when the Ca^2+^ concentration in the cytoplasm reaches a certain level (Cai et al., 2016), which can lead to Ca^2+^ overload (Nyberg and Espinosa, 2016). This Ca^2+^ overload in turn results in the abnormal accumulation of unfolded/misfolded proteins in the ER lumen by interfering with Ca^2+^-dependent chaperones (Nyberg and Espinosa, 2016), and subsequently, ERS is activated (Zhang et al., 2019). Besides, Ca^2+^ overload and ROS accumulation can affect each other (Hempel and Trebak, 2017). On the one hand, ROS can increase the permeability of the ER membrane and cause the Ca^2+^ reserves in the ER to be released into the cytoplasm, which often induces Ca^2+^ overload (Zhang et al., 2019). On the other hand, this Ca^2+^ overload can lead to the enhanced generation of ROS (Brookes et al., 2004). Mitochondrial Ca^2+^ can often be taken up when the cytosolic Ca^2+^ concentration is increasing (Takekawa et al., 2012). Eventually, Ca^2+^ stimulates the mitochondria to produce superoxide O_2_•^−^ by regulating the tricarboxylic acid (TCA) cycle and ETC enzymes. O_2_•^−^ then rapidly gets converted to ROS (Hempel and Trebak, 2017). Drug-induced ERS, which acts as a double-edged sword, decides the survival or death of the solid tumor cells. Adaptive ERS can cause solid tumor cell survival and drug resistance.

### The Main Response of ERS: UPR

ER plays an important role in protein folding, regulating lipid synthesis, and maintaining Ca^2+^ homeostasis (King and Wilson, 2020). However, when an imbalance occurs in the ER functioning between the demands and the capacities, ERS is often caused by the accumulation and aggregation of unfolded/misfolded proteins. To restore normal ER functioning, UPR is initiated in it. UPR plays a significant role in integrating the pro-death or pro-survival signals of the solid tumor cells (Lin et al., 2019). UPR is often responsible for the tumor cell pro-survival status by promoting protein folding in the solid tumors (Tian et al., 2017). The inositol requiring enzyme 1 (IRE1α), the protein kinase RNA-activated (PKR)-like ER kinase (PERK), and the activating transcription factor 6 (ATF6), which are transmembrane proteins on the ER, are also important sensors of UPR (Urra et al., 2016).

The activation of the above sensors is controlled by a key ER chaperone known as the glucose-regulated protein 78 (GRP78, also referred to Bip or HSPA5) (Hetz et al., 2015; Avril et al., 2017; Luo et al., 2018). GRP78 is separated from the three sensors when unfolded/misfolded proteins accumulate in the lumen of the ER (Li L. et al., 2015; Avril et al., 2017; Zhou et al., 2019), and subsequently, GRP78 is released into the lumen of the ER to bind with the unfolded/misfolded proteins, which promotes protein folding and trafficking (Li L. et al., 2015). Therefore, the high level of GRP78 in the ER lumen is a hallmark of ERS occurrence (Abdel Malek et al., 2015; Gifford et al., 2016). GRP78, as a pro-survival factor, plays an indispensable role in the process of solid tumor cells against ERS (Luo et al., 2018; Ryabaya et al., 2018).

PERK undergoes trans-autophosphorylation after its dissociation from GRP78 and phosphorylates the downstream eukaryotic initiation factor 2α (eIF2α) (Avril et al., 2017). The phosphorylation of eIF2α not only inhibits protein translation but also upregulates the expression of activating transcription factor 4 (ATF4) (Avril et al., 2017). ATF4 activates C/EBP homologous protein (CHOP) (Li Y. et al., 2018). It has been reported (Wang et al., 2017; B'Chir et al., 2014) that the activation of CHOP *via* ERS can induce autophagy or apoptosis.

IRE1α undergoes trans-autophosphorylation after its dissociation from GRP78. The activated IRE1α binds with x-box binding protein-1 (XBP1) and cleaves the introns to form XBP1s. Subsequently, the protein folding–associated proteins are activated by XBP1s (Avril et al., 2017). Furthermore, XBP1s activate the other nuclear genes, including the ER–associated protein degradation (ERAD) genes (Li et al., 2019).

ATF6 is initially transported to the Golgi apparatus after its dissociation from GRP78. In the Golgi apparatus, ATF6 is cleaved by S1P and S2P proteases and forms the N-terminal cytoplasmic fragment with ATF6 function as an activated transcription factor that reaches the nucleus and promotes the expression of several genes, such as GRP78 (Wu et al., 2007).

### ERS-Mediated Drug Resistance Pathways

Three sensor-mediated pathways of UPR lead to changes in the levels and activities of key regulatory factors that play a significant role in integrating the pro-death or pro-survival signals of solid tumor cells (Avril et al., 2017). Intrinsic ERS of the solid tumor cells can be induced by several ways, including apoptosis inhibition pathway, protective autophagy pathway, ABC transporters pathway, Wnt/β-catenin pathway, and noncoding RNA.

#### Apoptosis Inhibition

Apoptosis, which refers to a unique way of programmed cell death (PCD), is a process that occurs under a series of gene regulations (Tower, 2015; Ou et al., 2017). Apoptosis includes the extrinsic and intrinsic pathways (Iurlaro and Muñoz-Pinedo, 2016). The extrinsic pathway encompasses the death receptor pathway (Huang et al., 2018), while the intrinsic pathway encompasses the mitochondria-dependent and the ERS-mediated pathways (Iurlaro and Muñoz-Pinedo, 2016; Huang et al., 2018). Apoptosis induction is the most important approach of drug therapy for solid tumors (Goldar et al., 2015). ERS-mediated apoptosis will be induced if drugs cause ER dysfunction and result in a strong ERS. If any link in the process of drug-mediated apoptosis is impaired, apoptosis inhibition may lead to the survival of solid tumor cells, which will make the cells resistant to the drug (Goldar et al., 2015). It has been reported (Makhov et al., 2018; Tibullo et al., 2018) that ERS-mediated apoptosis inhibition is mainly related to the expression of antiapoptotic genes such as NF-κB, the antiapoptotic members of Bcl-2 family, and heme oxygenase-1 (HO-1).

##### NF-κB

**FIGURE 1:**
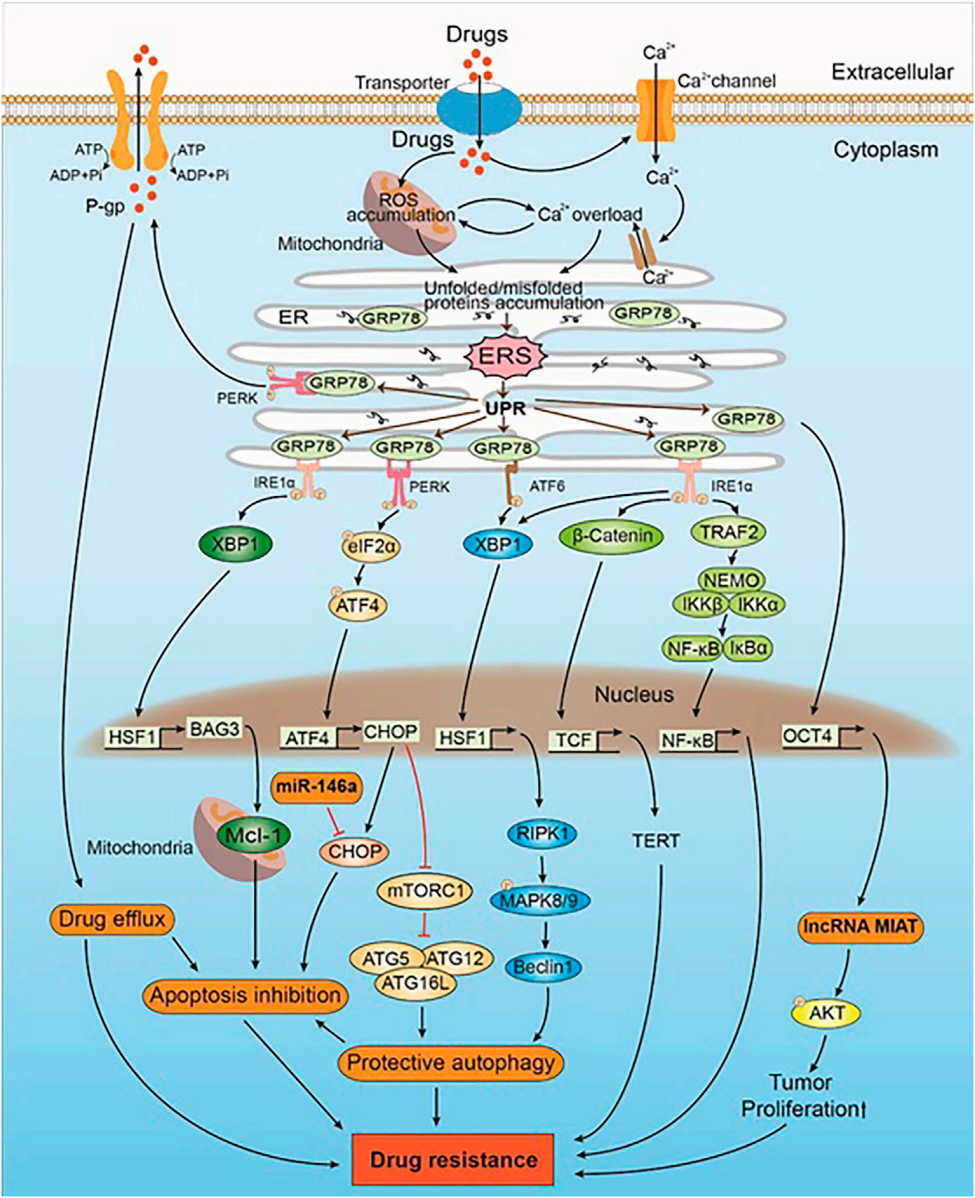
The mechanism of drug-induced ERS caused drug resistance in intrinsic direction. Drugs induce ERS by ROS accumulation and Ca^2+^ overload. UPR is activated when ERS occurs. GRP78, which originally binds to the UPR sensors (i.e., PERK, IRE1α, and ATF6), inhibits their activation. GRP78 can upregulate the expression of lncRNA MIAT and Akt by positively regulating the expression of OCT4. AKT phosphorylation leads to drug resistance by promoting the proliferation of tumor cells. PERK trans-autophosphorylates after the dissociation of GRP78 and phosphorylates downstream eIF2α, which in turn activates CHOP by regulating the expression of ATF4. CHOP promotes protective autophagy, which leads to drug resistance by inhibiting mTORC1 and promoting the expression of the ATG5–ATG12–ATG16L complex. CHOP is also a mediator of apoptosis. miR-146a induces drug resistance by inhibiting CHOP-mediated apoptosis. Some intracellular drugs are pumped out of the tumor cells by P-gp that is increased by PERK, which in turn reduced drug-induced solid tumor cell apoptosis and led to drug resistance. IRE1α is activated after dissociating from GRP78, and the downstream XBP1 upregulates the expression of HSF1. HSF1 upregulates the expression of BAG3, which stabilizes the expression of antiapoptotic protein Mcl-1, thereby inhibiting apoptosis and leading to drug resistance. In addition, both IRE1α and ATF6 promote the expression of XBP1 and downstream HSF1. HSF1 promotes the expression of Beclin-1 through the RIPK1-MAPK8/9 axis, inducing protective autophagy that leads to drug resistance. IRE1α also triggers the Wnt signaling pathway and NF-κB to promote solid tumor cell survival, which subsequently leads to drug resistance.

NF-κB, a transcription factor, is important for solid tumor development and progression (Li F. et al., 2015; Patel et al., 2018). The NF-κB protein family, which is a pleiotropic nuclear transcription factor family, includes p50, p52, p65 (RelA), c-Rel, and RelB, which share a common N-terminal Rel homology domain (RHD) for DNA binding (Hayden and Ghosh, 2008). Inactivated NF-κB dimers are associated with IκB proteins, which are localized in the cytoplasm (Hayden and Ghosh, 2008). Under multiple stimuli, a trimeric IκB kinase (IKK) complex phosphorylates other IκB family members (Betzler et al., 2020). The phosphorylated IκB proteins undergo proteasomal degradation *via* polyubiquitinylation, which promotes the release of NF-κB dimers and the nuclear translocation (Hayden and Ghosh, 2008; Betzler et al., 2020). Abnormal activation of NF-κB leads to antiapoptosis and the upregulation of pro-tumorigenic genes in the sunitinib-treated refractory phenotype of renal cell carcinoma (Makhov et al., 2018). IRE1α-mediated ERS response promotes the NF-κB transcriptional survival program mediated by TRAF2 to protect the solid tumor cells from cell death (Makhov et al., 2018; Figure 1). The activation of PERK sensitizes IRE1α, triggers the NF-κB pathway, leads to the expression of the apoptosis inhibitor protein XIAP, and causes drug resistance in imiquimod-treated melanoma cells (El-Khattouti et al., 2016).

##### Antiapoptotic Genes of the Bcl-2 Family

The B-cell leukemia/lymphoma-2 (Bcl-2) family is the key regulator in the process of apoptosis (Siddiqui et al., 2015). According to the different effects of the Bcl-2 family members on apoptosis regulation, they are divided into antiapoptotic and proapoptotic proteins (Su et al., 2014; Kang et al., 2018). Antiapoptotic proteins include Bcl-2, Bcl-xl, and Mcl-1, whereas proapoptotic proteins include Bad, Bim, and Noxa. Bcl-2, which is mainly located in the inner or the outer membrane of the mitochondria and the ER, plays an important role in mitochondria-mediated apoptosis and also influences ERS-mediated apoptosis (Lei et al., 2015; Liu et al., 2016; Xu et al., 2018). The antiapoptotic function of Bcl-2 is attributed to its regulation of intracellular Ca^2+^ (Xu et al., 2018). Bcl-2 downregulates the Ca^2+^ signal transduction of ER and mitochondria by interacting with the Ca^2+^ release channel IP3R in the ER and the Ca^2+^ intake channel, that is, voltage-dependent anion channel 1 (VDAC1), in the mitochondria. Hence, Bcl-2 can protect the cells from apoptosis by ERS/mitochondrial to reduce the cytotoxicity of antitumor drugs and result in drug resistance (Xu et al., 2018). In addition, insulin resistance (IR) can cause 5-fluorouracil resistance in hepatocellular carcinoma by activating the ERS–PERK pathway and Bcl-2 upregulation (Liu et al., 2016). The antiapoptotic protein Mcl-1 is located in the mitochondria and often leads to drug resistance in solid tumors because of its high expression. In mutant KRAS colon cancer cells, the high expression of Mcl-1 is stabilized by the Bcl-2–associated athanogene domain 3 (BAG3), which is upregulated by the heat shock factor 1 (HSF1) and downstream of XBP1, and HSF1 can cause resistance to the HSP90 inhibitor AUY922 (Wang et al., 2018; Figure 1).

##### HO-1

HO-1, which is widely distributed in various mammalian cells, plays a key role in anti-oxidation, anti-inflammation, and antiapoptosis (Kim et al., 2011). HO-1 is very important for the survival mechanism and maintains homeostasis by cleaving heme to form biliverdin, carbon monoxide (CO), and iron (Kim et al., 2011; Tibullo et al., 2018). HO-1 is often induced by heat shock reaction (HSR) and other intracellular stress factors. However, ERS is an important inducing factor in intracellular stress. The expression of HO-1 might be related to PERK-dependent Nrf2 activation (Kim et al., 2007). The overexpression of HO-1 is found in many solid tumor cells, such as melanoma and neuroblastoma, and is thought to be closely related to drug resistance (Barbagallo et al., 2015; Tibullo et al., 2018).

#### Protective Autophagy

Autophagy is a kind of PCD which is different from apoptosis (Ojha et al., 2015; Ou et al., 2017). Autophagy can not only induce cell death under severe stress but also promote cell survival by devouring the damaged organelles and providing new energy for the cells (Jiang et al., 2019). The latter is called protective autophagy. ERS-related autophagy is activated in many solid tumors during therapy, and it can induce protective autophagy and increase autophagy flux to protect cells from death (Ji et al., 2016; Cheng et al., 2018; Liu et al., 2018). ERS-related protective autophagy signaling mainly includes mTORC1 inhibition, Beclin-1, ATG5–ATG12 complex formation, and LC3 conversion.

##### mTORC1 Inhibition

The mammalian target of rapamycin (mTOR), which is a master regulator of cell growth and metabolism (Appenzeller-Herzog and Hall, 2012), is a conserved serine/threonine kinase that binds to several proteins to form two different complexes: mTORC1 and mTORC2 (Kawasaki et al., 2018). mTORC1 plays a negative regulatory role in autophagy by inhibiting the formation of the ULK complex containing various ATG core proteins necessary for autophagy. Inhibition of mTORC1 by CHOP promotes autophagy downstream of PERK in salinomycin-treated non-small cell lung cancer (NSCLC) cells (Li et al., 2013; Figure 1).

##### Beclin-1

Beclin-1, which is a core molecule in autophagy, mediates the localization of autophagy proteins in autophagosomes and participates in the formation of autophagosomes to facilitate their maturation (De et al., 2019; Cheng et al., 2017). Beclin-1 is involved in the formation of three phosphatidylinositol 3-kinase (PtdIns3K) complexes, namely, ATG14 complex, UVRAG complex, and Rubicon complex (Yang and Klionsky, 2010). Among them, ATG14 and UVRAG are PtdIns3K complexes needed for autophagy, which are conducive to promote autophagy. Zhou et al. (2019) reported that the expression of Beclin-1 can be activated by PERK-dependent ATF4 to promote protective autophagy, which is responsible for the resistance of hepatocellular carcinoma to sorafenib. Autophagy is inhibited when Beclin-1 binds to Bcl-2/Bcl-xl and destroys Beclin-1–related PtdIns3K complexes. The phosphorylation of Bcl-2 leads to the disintegration of the Beclin-1/Bcl-2 complex through the IRE1α–ATF2–JNK pathway, which initiates the formation of Beclin-1–related complex and activates protective autophagy. The phosphorylation of BCL2L11/BIM is mediated by MAPK8/JNK1 or MAPK9/JNK2, which is activated by the high expression of receptor-interacting protein kinase 1 (RIPK1) by the IRE1α-XBP1-HSF1 axis (Luan et al., 2015). This activation results in BECN1/BCL2 separation and activates protective autophagy in tunicamycin-treated melanoma cells (Luan et al., 2015; Figure 1). “Nucleotide-binding, lots of leucine-rich repeats-containing protein member X1” (NLRX1)-Tu translation elongation factor mitochondrial (TUFM) protein complex recruits Beclin-1 into the mitochondria to facilitate its polyubiquitination and interferes with its interaction with Rubicon to promote protective autophagy in EGFR-targeted mAb (such as cetuximab) therapy for head and neck squamous cell carcinoma (Lei et al., 2016).

##### ATG5–ATG12 Complex and LC-3 Transformation

ATG5–ATG12 complex and LC-3 are involved in the formation and extension of the membrane structure of the autophagy precursor (Döring and Prange, 2015; Kharaziha and Panaretakis, 2017). The expression of ATG12 is upregulated by ATF4 *via* PERK and its downstream signal. The expression of ATG5 is induced by CHOP *via* pentoxifylline-induced IRE1α in pentoxifylline-resistant melanoma (Sharma et al., 2016). The expression levels of ATG5 and ATG12 are improved to benefit the formation of the ATG5–ATG12 complex, which can initiate the closure of the autophagosome membrane. The conversion of LC3 is promoted by CHOP to further initiate the formation of autophagosome membrane and induce protective autophagy in solid tumors treated with antitumor drugs (Yu et al., 2017). For example, in the study of cisplatin-induced drug resistance in lung cancer cells, it was found that 3-MA and CQ inhibit the production of the autophagy marker LC3 and enhance cisplatin-induced cell death (Shi et al., 2016). Similarly, Yu et al. (2017) reported that salinomycin-induced autophagy in glioma is mediated by ERS, and this autophagy protects the glioma cells from death. Besides, ATF4 regulates the expression of LC3 and promotes autophagy by binding to the promoter of LC3B in methotrexate-resistant choriocarcinoma cells (Shen et al., 2015).

#### ABC Transporters

The ATP-binding cassette (ABC) transmembrane transporter superfamily, which is a drug efflux transporter, is closely related to solid tumor resistance (Alexa-Stratulat et al., 2019). The ABC transporters contain two ATP binding domains and multiple drug binding domains (Thomas and Tampé, 2018). They facilitate the transmembrane transport of drugs and other molecules through active transport. These transporters collaborate with ERS to promote drug resistance. This manuscript mainly deals with P-gp, ABCG2, and MRP1.

##### P-gp

P-glycoprotein (P-gp), also known as ABCB1, is located in the cell membrane as a member of the ABC superfamily. P-gp is the most widely studied multidrug resistance protein in the family of ABC transporters (Silva et al., 2015; Ge et al., 2017). The protein is an energy-dependent drug pump that protects cells from harmful foreign molecules (Wang et al., 2004; Li S. et al., 2018). The pump is widely distributed in normal human tissues, such as the mucosal cells of the small intestine and the endothelial cells of the blood–brain barrier (Hano et al., 2018). P-gp is naturally present in the body tissue, but solid tumor cells can often activate and upregulate it through cellular stress. Studies have shown (Li S. et al., 2018; Liu et al., 2016) that P-gp can be upregulated by insulin resistance (IR). IR-induced P-gp upregulation promotes the drug resistance of solid tumor cells by pumping drugs out and functioning as an ER chaperone protein that participates in the ERS by directly or indirectly transporting unfolded/misfolded proteins. This process causes the hepatocellular carcinoma to become resistant to a variety of chemotherapeutic agents, including cisplatin and doxorubicin (Li L. et al., 2015; Liu et al., 2016).

##### ABCG2

ABCG2 is a member of the ABC superfamily and is known as the breast cancer resistance protein (BCRP) gene, which is closely related to the emergence of solid tumor resistance (Chen et al., 2018). The ABCG family has its own structural characteristics, which are different from those of the other members of the ABC superfamily. The ABCG family members have only one ATP-binding domain at the front end of the transmembrane region. In plasma cells, the expression level of ABCG2 becomes elevated under ERS (Chen et al., 2018). ABCG2 is associated with oxaliplatin resistance in LoVo colorectal cancer by pumping the drugs out (Chen et al., 2018).

##### MRP1

Among the ABC transporters related to multidrug resistance, multidrug resistance–associated protein-1 (MRP1) plays a very important role in the drug resistance of solid tumors. MRP1 protects the cells by pumping drugs out to achieve sublethal levels. The protein consists of three transmembrane domains, namely, TMD0, TMD1, and TMD2. Besides, it also contains two nucleotide-binding domains (NBD1 and NBD2) in the cytosol (Lu et al., 2015). MRP1 causes drug efflux, which lowers the intracellular drug concentration and enables the solid tumor cells to survive. Salaroglio et al. (2017) found that PERK/Nrf2 axis upregulates MRP1, which leads to the resistance of the colorectal cancer cells to platinum drugs such as oxaliplatin.

#### Wnt/β-Catenin Signaling Pathway

The Wnt signaling pathway involves the process of the Wnt protein activating the intracellular signaling by binding to the associated receptors (Rao and Kühl, 2010). The Wnt signaling pathway is closely related to the pathogenesis of many human diseases, besides influencing the development of drug resistance (Pujari et al., 2016; Rodvold et al., 2017). The pathway is divided into the classical and nonclassical Wnt signaling pathways. The classical Wnt signaling pathway is mediated by *β*-catenin, which is a core member and is involved in various cellular processes such as growth and differentiation (Rodvold et al., 2017). In the presence of the Wnt signal, disheveled (DSH) protein is activated after the Wnt protein binds to the frizzled receptor on the cell surface. The activated DSH protein enhances the phosphorylation of GSK-3β, which results in the accumulation of unphosphorylated *β*-catenin in the cytoplasm. This *β*-catenin is subsequently transported into the nucleus and binds to the TCF/LEF to form a complex that promotes changes in the transcription mechanisms. Transmissible ER stress (TERS) drives the Wnt signal through the activation of *β*-catenin *via* IRE1α (Rodvold et al., 2017). *β*-catenin can transcriptionally activate telomerase reverse transcriptase (TERT), which offers cell protection in prostate cancer cells treated with bortezomib (Rodvold et al., 2017; Figure 1). ER+ breast cancer is a common type of breast cancer, and tamoxifen has been used in its treatment for a long time (Zhu et al., 2019). However, tamoxifen resistance is often present in ER+ breast cancer. Past research has suggested (Pujari et al., 2016) that tamoxifen-treated breast cancer cells induced the formation of AKT–GRP78 complex, which resulted in the phosphorylation of AKT at Thr308. The activated AKT subsequently phosphorylated its downstream substrate GSK-3β at Ser9, which in turn activated the downstream Wnt signal and caused the breast cancer cells to survive (Pujari et al., 2016).

#### Noncoding RNA

Noncoding RNA (ncRNA) denotes an RNA that cannot encode a functional protein commonly. Studies have shown (Yao et al., 2020; Tan et al., 2018) that ncRNA can regulate the survival and death of solid tumor cells *via* ERS. Noncoding miRNA and lncRNA are especially associated with ERS-mediated drug resistance in solid tumor cells (Wei et al., 2019). Slack and Chinnaiyan (2019) reported that ncRNA regulates the development of solid tumor cells by controlling gene expression at transcriptional or posttranscriptional levels or by epigenetic regulation, thus determining the resistance of solid tumor cells to antitumor drugs in diverse pathways (Slack and Chinnaiyan, 2019). The expression of lncRNA MIAT is upregulated by GRP78 and its downstream Akt by positively regulating the expression of OCT4 (Yao et al., 2020; Figure 1). Akt phosphorylation leads to 5-fluorouracil resistance in breast cancer cells by promoting the proliferation of solid tumor cells (Yao et al., 2020). miR-146a can inhibit CHOP, which is a mediator of ERS-induced apoptosis (Kudo, 2003). The downregulating expression of CHOP leads to apoptosis inhibition. Significantly, Tan et al. (Tan et al., 2018) found that miR-146a induces cisplatin resistance in lung cancer cells by binding to CHOP 3′UTR to inhibit CHOP (Figure 1). Furthermore, another study indicated (Grzywa et al., 2020) that vemurafenib promotes the expression of miR-410-3p by inducing 12 transcription factors downstream of ERS in melanoma. The overexpression of miR-410-3p results in the transformation of melanoma cells into an invasive phenotype, thereby causing their resistance to vemurafenib (Grzywa et al., 2020). In summary, miRNA or lncRNA can regulate the survival and death of solid tumor cells *via* ERS regulatory factors, which is the key to solid tumor resistance.

## Extrinsic Drug-Induced ERS and Drug Resistance in Solid Tumors

TME, which acts as a “protective net” for solid tumor cells, includes several surrounding stromal cells, endothelial cells, and different types of immune cells (Varol, 2019). Among them, immune cells often play a pertinent role in anti-solid tumors. The innate (such as DCs and MDSCs) and adaptive immune cells (such as T cells) contribute to solid tumor progression when present in the TME (Hinshaw and Shevde, 2019). In the TME, immunosuppression is a common event that is triggered by the inhibition of activated immune cells or by the production of immunosuppressive cells (Lima et al., 2018). Thus, anti-solid tumor therapy is essential for the process of immunosuppression. Different conditions in the TME may disrupt the load of newly synthesized proteins and trigger ERS in tumor cells and their surrounding immune cells. ERS in immune cells can cause immunosuppression by impairing their function and even promoting their death (Cubillos-Ruiz et al., 2017).

### Drug-Induced ERS in Immune Cells

Antitumor drugs can promote ERS in immune cells by inducing oxidative stress (OS) and high ROS accumulation (Cubillos-Ruiz et al., 2017). In DCs, high ROS levels lead to intracellular lipid peroxidation and expression of 4-hydroxynonenal (4-HNE), which induces ERS. In MDSCs, high ROS accumulation also initiates ERS. In general, the TME is often characterized by limiting concentrations of oxygen, glucose, and other nutrients (Young et al., 2013). In T cells, glucose restriction induces ERS by impairing optimal N-linked protein glycosylation (Song et al., 2018). Meanwhile, tumor-derived or exogenous cholesterol leads to lipid metabolism disorder, which in turn triggers ERS in T cells (Picarda et al., 2019).

### ERS-Related Immunosuppression

Immunosuppression, which is an important factor in the development and metastasis of solid tumors, is closely related to antitumor drug resistance (Pommier et al., 2018). Here, we have chiefly discussed how DCs, MDSCs, and T cells trigger ERS under adverse conditions in the TME, thereby leading to immunosuppression.

**FIGURE 2:**
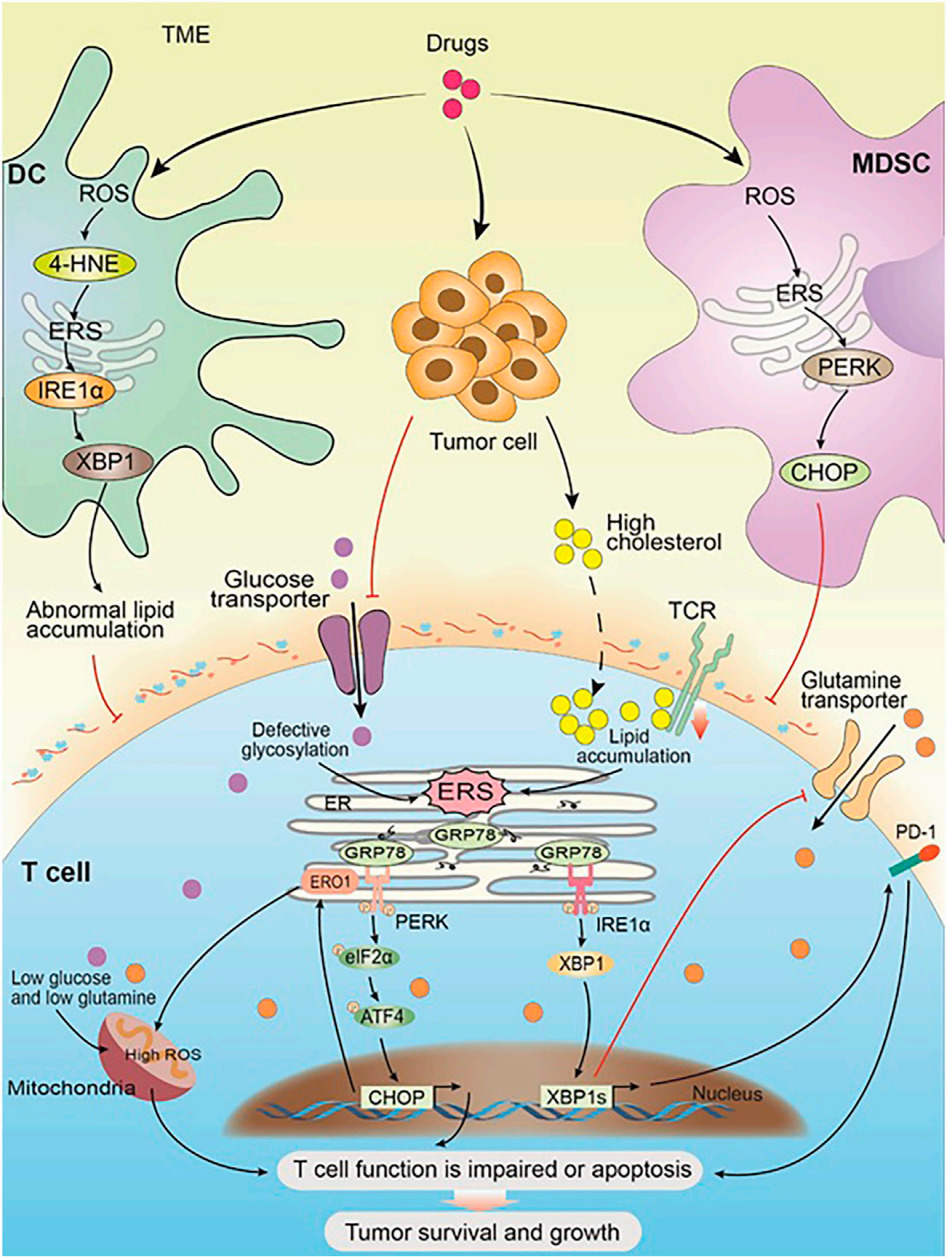
ERS within immune cells promotes solid tumor cell survival and progression. Drug and the progress of tumor cause changes in the TME and trigger ERS in the immune cells. In DC, the high expression of ROS leads to an increase of 4-HNE, which in turn induces ERS and its downstream IRE1α-XBP1 arm, resulting in abnormal lipid accumulation, which inhibits the antigen presentation of tDCs to T cells. In MDSC, the high expression of ROS activates ERS, and its downstream PERK-CHOP arm inhibits the normal function of T cells. Glucose restriction in solid tumor microenvironment triggers ERS by impairing optimal N-linked protein glycosylation in T cells, which in turn inhibits glutamine transporter–mediated glutamine influx. Both glutamine and glucose flow into the mitochondria get reduced as a result, and the mitochondrial respiratory function and the T-cell function get impaired. Solid tumor-derived or exogenous cholesterol leads to lipid metabolism disorders in CD8^+^T cells, triggering the high expression of internal ERS and XBP1, which in turn promotes the high expression of PD-1 and leads to T-cell apoptosis. Meanwhile, cholesterol inhibits TCR signaling by binding to the transmembrane region of the TCRb chain and disrupting TCR clustering, which inhibits the normal function of T cells and leads to immunosuppression. Chronic PERK induces the downstream target ERO1 through the ATF4-CHOP axis, leading to a large amount of ROS accumulation and impaired mitochondrial function. Impaired T-cell function or apoptosis promotes the survival and development of solid tumor cells.

DCs serve as the link between innate and adaptive immune systems by transmitting information in the form of antigens to adaptive immune cells. DCs activate T cells by cross-presenting exogenous antigens (Fu and Jiang, 2018). ERS in DCs hinders this antigen presentation and affects the normal functioning of the T cells. Velez et al. (2011) discovered that some cytotoxic drugs can induce OS and then produce 4-HNE. For example, high ROS levels can cause intracellular lipid peroxidation and expression of 4-HNE in ovarian tumor-infiltrating dendritic cells (tDCs). 4-HNE disrupts ER homeostasis and activates UPR by acting on ER chaperones. UPR activates its downstream IRE1α-XBP1, which in turn inhibits the antigen presentation of tDCs to T cells and promotes immunosuppression (Cubillos-Ruiz et al., 2015; Figure 2).

Furthermore, the proliferation of MDSCs plays an important role in anti-solid tumor immune escape (Mohamed et al., 2020). ERS in MDSCs can also affect the normal functioning of the T cells. The expression of PERK is elevated in human malignant solid tumors, and its downstream CHOP is involved in MDSC activity (Mohamed et al., 2020). The high expression of CHOP is directly related to the immune response ability of the T cells (Mohamed et al., 2020). Lower NRF2 signaling in PERK-deficient MDSCs can initiate ROS accumulation and block mitochondrial respiration, which can in turn reprogram the MDSCs into immune-stimulating cells and promote anti-solid tumor immunity (Mohamed et al., 2020; Figure 2).

ERS in T cells leads to their dysfunction and even death. T-cell dysfunction is seen in most of the tumor patients, and it is an important mechanism of immunosuppression (Cao et al., 2019). T cells include cytotoxic T cells (CD8^+^ T cells) and helper T cells (CD4^+^ T cells). CHOP, which is a downstream sensor of severe ERS, is the major negative regulator of the effector function of CD8^+^ T cells in solid tumors (Cao et al., 2019). Chronic PERK induces the downstream target ER oxidoreductase 1 (ERO1) through the ATF4-CHOP axis (Hurst et al., 2019). The overexpression of ERO1 leads to the accumulation of ROS, which is a marker of mitochondrial failure (Hurst et al., 2019; Figure 2). For example, in the TME of ovarian tumor, glucose restriction induces ERS by impairing the optimal N-linked protein glycosylation in T cells. Subsequently, XBP1s present downstream of IRE1α inhibits glutamine transporter–mediated glutamine influx, which is necessary to sustain mitochondrial respiration under glucose deprivation, thereby resulting in impaired mitochondrial respiratory function of T cells and promoting immunosuppression (Song et al., 2018). Tumor-derived or exogenous cholesterol leads to lipid metabolism disorders that trigger ERS and induce XBP1 overexpression, which upregulate the expression of programmed death protein PD-1 and initiate T-cell apoptosis (Picarda et al., 2019; Figure 2). Meanwhile, cholesterol inhibits TCR signaling by binding to the transmembrane region of the TCRb chain and disrupting TCR clustering, which inhibits the normal functioning of the T cells and leads to immunosuppression (Picarda et al., 2019; Figure 2).

In summary, ERS in the innate (DCs and MDSCs) and adaptive (such as T cells) immune cells promotes immunosuppression and facilitates tumor survival, which leads to drug resistance.

## Concluding Remarks and Future Perspectives

At present, drug resistance is the main problem faced during the treatment of solid tumors. ERS is attributed to the disruption of ER functioning by various stimuli. UPR is an adaptive response of ERS to the accumulation of unfolded/misfolded proteins. Drug-induced changes in solid tumor survival conditions activate ERS and UPR. UPR regulates the downstream pro-survival or pro-apoptotic signals through three sensors. Several studies have shown that the pro-survival signal mediated by ERS plays an important role in drug resistance in antitumor therapy. The mechanism of ERS in drug resistance is complex and not fully understood. In this review, we have shown that drug-induced ERS is associated with intrinsic (apoptosis inhibition, protective autophagy, ABC transporters, Wnt/β-catenin signaling pathways, and noncoding RNA) and extrinsic (immunosuppression) solid tumor adaptation mechanisms.

The targeted regulation of ERS-related regulatory factors and effectors and combining them with antitumor drugs are useful in improving drug sensitivity. For example, since the HSF1/BAG3/Mcl-1 axis can protect mutated KRAS colon cancer cells from AUY922-induced apoptosis, it is of potential significance to target this axis for improving the efficacy of AUY922 in colon cancer treatment (Wang et al., 2018). NF-κB inhibition enhances imiquimod-induced apoptosis of the melanoma cells and reverses chemotherapy resistance (El-Khattouti et al., 2016). miRNA 146a promotes cisplatin resistance in lung cancer cells by targeting CHOP (Tan et al., 2018). Tan et al. (2018) hypothesized that miR-146a is a potential therapeutic target for lung cancer cells exhibiting multiple drug resistance. The high expression of XBP1 can protect the cells from death in tamoxifen-treated breast cancer (Ming et al., 2015). A new chemical drug, STF-083010, can reverse this resistance by inhibiting the IRE1/XBP1 axis (Ming et al., 2015). In addition, since ERS-mediated protective autophagy is an important cause of drug resistance in solid tumor cells, the combination of antitumor drugs and autophagy inhibitors is effective in reversing this resistance. For example, targeted autophagy increases the sensitivity of BRAF mutant thyroid cancer to vemurafenib (Wang et al., 2017). Targeted autophagy enhances the apatinib-induced apoptosis of colorectal cancer cells through ERS (Cheng et al., 2018).

In conclusion, it is of great significance to study ERS-mediated drug resistance in solid tumor cells to explore combination therapy and develop new drugs. Furthermore, it is conducive for the development of translational medicine by providing insightful theories. Augmenting our understanding of this mechanism is also likely to be helpful in reversing the drug resistance of solid tumor cells and in opening new avenues for clinical solid tumor drug therapy.
